# Highly sensitive plasmonic paper substrate fabricated via amphiphilic polymer self-assembly in microdroplet for detection of emerging pharmaceutical pollutants

**DOI:** 10.1186/s40580-024-00420-x

**Published:** 2024-03-29

**Authors:** Mirkomil Sharipov, Sarvar A. Kakhkhorov, Salah M. Tawfik, Shavkatjon Azizov, Hong-Guo Liu, Joong Ho Shin, Yong-Ill Lee

**Affiliations:** 1 Anastro Laboratory, Institute of Basic Science, Changwon National University, Changwon, 51140 Republic of Korea; 2 School of Mechanical Engineering, Yonsei University, 50 Yonsei-ro, Seodaemun-gu, Seoul, 03722 Republic of Korea; 3 Department of Petrochemicals, Egyptian Petroleum Research Institute, Cairo, 11727 Egypt; 4 Department of Pharmaceutical Sciences, Pharmaceutical Technical University, Tashkent, 100084 Republic of Uzbekistan; 5 Key Laboratory for Colloid and Interface Chemistry of Education Ministry, Shandong University, Jinan, 250100 PR China; 6 Division of Smart Healthcare, College of Information Technology and Convergence, Pukyong National University, Busan, 48513 Republic of Korea

**Keywords:** Surface-enhanced Raman scattering, Air/liquid interface, Microdroplet, Self-assembly, Emerging pollutants

## Abstract

We report an innovative and facile approach to fabricating an ultrasensitive plasmonic paper substrate for surface-enhanced Raman spectroscopy (SERS). The approach exploits the self-assembling capability of poly(styrene-b-2-vinyl pyridine) block copolymers to form a thin film at the air-liquid interface within the single microdroplet scale for the first time and the subsequent in situ growth of silver nanoparticles (AgNPs). The concentration of the block copolymer was found to play an essential role in stabilizing the droplets during the mass transfer phase and formation of silver nanoparticles, thus influencing the SERS signals. SEM analysis of the morphology of the plasmonic paper substrates revealed the formation of spherical AgNPs evenly distributed across the surface of the formed copolymer film with a size distribution of 47.5 nm. The resultant enhancement factor was calculated to be 1.2 × 10^7^, and the detection limit of rhodamine 6G was as low as 48.9 pM. The nanohybridized plasmonic paper was successfully applied to detect two emerging pollutants—sildenafil and flibanserin—with LODs as low as 1.48 nM and 3.45 nM, respectively. Thus, this study offers new prospects for designing an affordable and readily available, yet highly sensitive, paper-based SERS substrate with the potential for development as a lab-on-a-chip device.

**Supplementary Information:**

The online version contains supplementary material available at 10.1186/s40580-024-00420-x.

## Introduction

Block copolymers (bCP) are polymers that consist of at least two different or immiscible polymers connected by covalent bonds. Szwarc was the first to synthesize amphiphilic bCPs using living anionic polymerization back in the 1950s [1]. Since then, various bCP, such as linear, graft, and branched copolymers, have been synthesized using different polymerization methods [2]. The discovery of bCPs’ ability to spontaneously self-assemble in the 1960s [3] revolutionized the field of polymer science and paved the road for their use to generate nanostructures [4, 5], such as micelles [6], nanorods [7], and polymersomes [8]. The ability of bCPs to self-assemble into various nanostructures is mainly governed by their molecular structure, composition, concentration, and response to external factors. Assembly of block copolymers at various interfaces, including air-water [9], oil-water [10], or solid-liquid [11], can generate innovative materials with adjustable shapes, structures, and properties.

In our previously reported work, we developed a novel strategy for the fabrication of composite thin films via the self-assembly of poly(styrene)-*b*-poly(vinyl pyridine) block copolymer (bCP) incorporating silver ions at the air-liquid interface [12]. The morphology of these films could be easily tuned and varied from nanowires to honeycomb-like structures depending on the concentration of the components. A similarly flexible approach to controlling the formation of silver nanoparticles in the desired fashion could be most interesting for developing a surface-enhanced Raman spectroscopy (SERS) substrate.

The SERS is a powerful vibrational spectroscopy technique that enables the enhancement of the Raman signal by many orders of magnitude, providing fine molecular fingerprints and allowing the detection of trace species [13]. In this regard, the development of highly sensitive SERS substrates, i.e., enabling extensive hot spot generation, has been a cornerstone for achieving extraordinary efficiency to the extent of distinguishing even single molecules [14, 15]. The uniform distribution of metal nanoparticles across the substrate in appropriate proximity to each other is pivotal, as it determines the ability of the SERS-sensitive regions to interact with the analytes [16]. Designing a facile and rapid method for the fabrication of a cost-effective SERS substrate that enables traces of target molecules to be detected continues to remain a major challenge, particularly in resource-limited situations.

Recently, paper substrates have become increasingly popular among researchers in the SERS community owing to the numerous advantages these substrates offer over their traditional counterparts [17, 18]. Paper is readily available, inexpensive, and biodegradable; in addition, its surface can be easily functionalized to vary its affinity toward analytes and solvents [19–21]. Apart from this, the porous structure of paper enables its application to microfluidics [22, 23], whereas its mechanical properties present unique opportunities for the collection of real-world samples [24]. Most importantly, the above-listed features can be achieved without sacrificing the sensitivity of the SERS technique, with a satisfactory enhancement factor (EF) ranging from 10^5^ to 10^8^ [18, 25, 26].

Gold and silver nanoparticles have been largely utilized as agents to enhance the Raman scattering in SERS mainly because of their stability and excellent enhancement properties [27, 28]. Metal nanoparticles can be fabricated via self-assembly [29], ink-jet printing [25], the deposition of presynthesized colloidal metal nanoparticles [30], surfactant-free synthesis [31], silver-complex ink [32], etc. Nevertheless, any alternative approach that would entail a more facile yet still efficient procedure devoid of disadvantages such as the cost and operating time remains attractive and worthy of investigation.

**Fig. 1:**
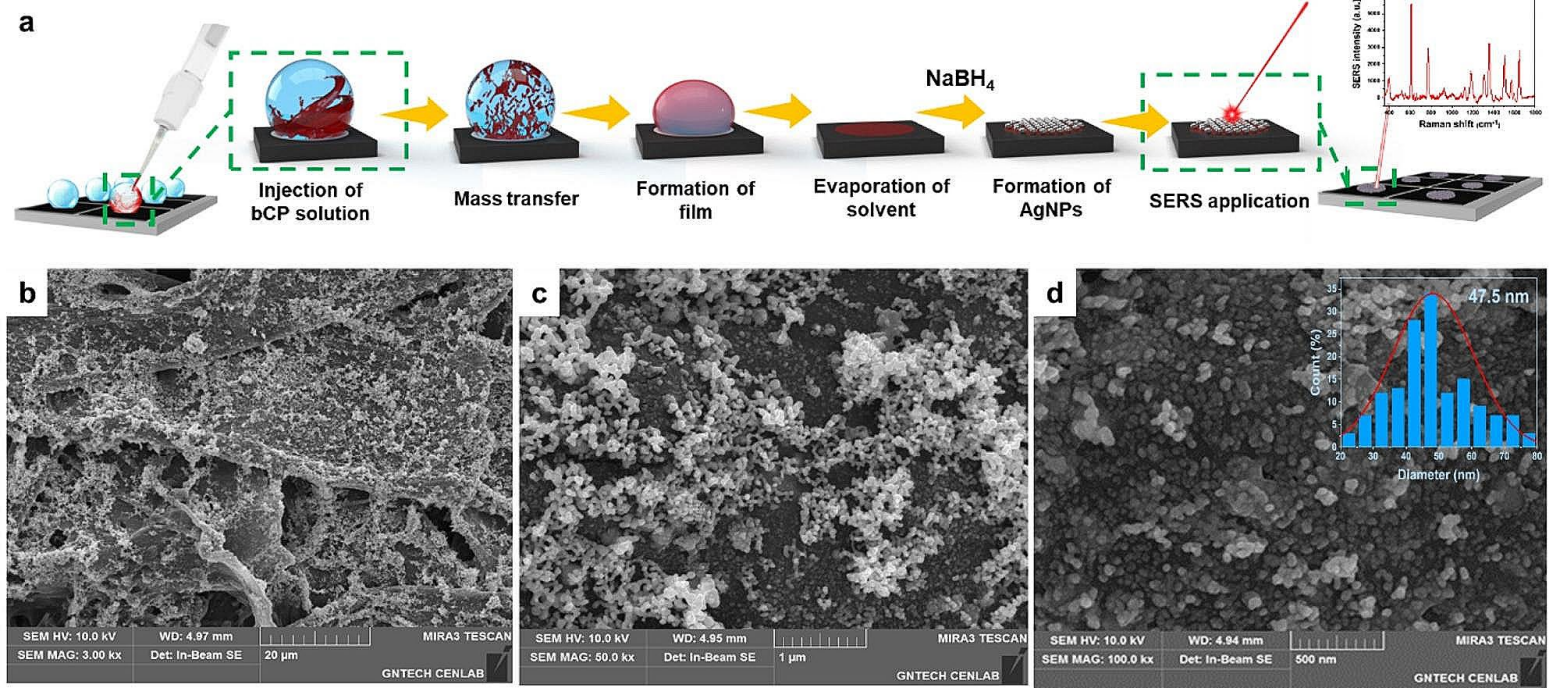
Schematic illustration of the preparation of an organic film containing silver nanoparticles at the microliter scale via the self-assembly of poly(styrene)-b-poly(vinyl pyridine) copolymer molecules at the air-liquid interface. (**a**) FE-SEM images of nanohybridized paper-substrate fabricated with 0.1 mg mL^− 1^ of bCP and 0.05 M AgNO_3_ at three different magnifications (**b**, **c**, and **d**). Inset: histogram presenting the size distribution of the silver nanoparticles

Here, we report a cost-effective, easy-to-operate, and environmentally friendly method for the in situ fabrication of a SERS substrate. The method exploits the self-assembly of a bCP in a single microdroplet to form a thin organic film doped with silver nanoparticles (AgNPs) on a paper surface for the first time. While numerous reports discuss the self-assembly of bCP in droplets, these droplets typically consist of emulsion or microfluidic droplets within a stable system [33–35]. We managed to achieve the use of exceptionally low volumes of components in the microliter range such that the entire self-assembly process occurs within the air/liquid interface of a single droplet deposited on paper substrate (Fig. 1a). This method does not require the preparation of colloidal metal nanoparticles prior to the fabrication, thereby avoiding complications related to their preparation and storage [36]. Wax-patterned office paper was used as a substrate because of its rough surface, availability, and biodegradability. The denser structure of the office paper compared to Whatman No.1 filter paper allowed the contact angle of the droplet to be increased, thus resulting in uniform fabrication devoid of the notorious “coffee ring” effect [37]. More importantly, the results showed that the bCP has a double function. The first is to promote the self-assembly of silver-loaded films, and the second is the stabilization of the microdroplet. These conditions led to the formation of uniform silver nanospheres with an average size of 47.5 nm. SERS measurements of rhodamine 6G (R6G) demonstrated that the developed substrates have good reproducibility in terms of reaching their limit of detection of 48.9 pM. The practicability of the developed sensor was demonstrated by using it for the detection of the emerging environmental pollutants sildenafil (SD) and flibanserin (FLBN) with calculated limits of detection of 1.48 nM and 3.45 nM, respectively.

## Methods/Experimental

### Materials

Poly(styrene-*b*-2-vinyl pyridine) (M_n_: 110,000-b-12,500) was purchased from Polymer Source, Inc. (Canada). Silver nitrate (AgNO_3_, ≥ 99.0%) was acquired from Sigma Aldrich. Sodium borohydride (NaBH_4_, ≥ 99.0%) was purchased from Fluka Analytical. Rhodamine 6G (99%) was purchased from Acros Organics. Sildenafil citrate (HPLC grade, > 98.0%) and flibanserin (HPLC grade, > 97.0%) were purchased from Tokyo Chemical Industry (TCI chemicals, Japan). Dimethylformamide (ACS grade, 99.8+ %) was purchased form Alfa Aeser. Chloroform (99%) was purchased from Samchun Chemical (South Korea). Conventional office paper (A4|75 g m^− 2^) was purchased from Hansol Copy, South Korea. Ultrapure water purified by the Milli-Q water purification system (Millipore Corp., MA, USA) was used throughout the experiments. All reagents were used without further purification or modification.

### Instrumentation

SERS measurements were performed on an EnSpectr R532-50 Enspectr Portable Raman Luminescent Spectrometer probe (Enhanced Spectrometry Inc, 560 South Winchester Blvd, #500 San Jose, USA) equipped with a 532-nm wavelength laser and a Jasco NRS-3300 Micro/Macro Raman spectrophotometer with a 532-nm laser. The morphology of the paper surface was studied by examining FE-SEM images captured with a CZ/MIRA I LMH microscope (Czech Republic). UV-Vis absorbance measurements were conducted on a V-670 spectrophotometer (Jasco Inc.). Contact angle of droplets were estimated using software ImageJ and “Contact_Angle” plugin. X-ray diffraction (XRD) was carried out on a Bruker D8 Discover diffractometer with Cu Kα radiation (λ = 0.1542 nm). Samples were fixed to a glass substrate for the analysis.

### Fabrication of plasmonic nanohybridized paper substrate

Wax-patterned multi-well paper sheets with designed hydrophilic wells with diameters of 4 mm were printed on a Xerox ColorQube 8570DN PS (Xerox Corporation Wilsonville, 97,070, OR, USA). The solid wax ink was deposited twice to increase the water-resistance of the wax barriers on the 200 × 200 mm A4 (210 × 297 mm) printing paper sheets (Hansol Paper, Jung-gu, Seoul, Korea). The paper sheets were wrapped in aluminum foil and placed on a hot plate (Daihan Scientific Co., Ltd.) for 30 s at 100 °C to melt the wax to allow for the thorough distribution thereof over the cellulose fibers of the paper, after which the diameter of the wells was reduced from the initial 4 mm to 1.5 mm.

In a typical experiment, 4 µL of AgNO_3_ of the desired concentration was drop-casted into the wells with a micropipette to form a droplet that occupies the entire surface of each well. Then, 2 µL of bCP dissolved in dimethylformamide (DMF) and chloroform (CHCl_3_) (0.1 mg mL^− 1^; V_DMF_/V_CHCl3_ = 3/2) was slowly injected into each droplet using a microsyringe. The paper substrate was then placed in the oven for 10 min at 30 °C to allow the solvents to gently evaporate. Evaporation of the solvent led to the formation of a thin copolymer film, the existence of which was divulged by the observed reflection of room light from the surface of the wells. Next, the paper substrate was immersed in an aqueous solution of NaBH_4_ (0.01 M) for 30 min. The color of the wells changed to dark yellow as a result of the reduction of the Ag^+^ to form AgNPs. Finally, the paper substrate was dried in the vacuum oven for 10 min at 65 °C and used immediately or within 48 h, during which time it was kept at 4 °C in glass vials filled with nitrogen and wrapped with aluminum foil.

### SERS measurements

SERS measurements were conducted on a R532-50 Enspectr Portable Raman Luminescent Spectrometer probe using a wavelength of 532 nm, a Raman shift range of 109 − 4050 cm^− 1^, and an integration time of 30 s. In a typical experiment, 5 µL of the analyte (R6G, SD, and FLBN) in different concentrations were drop-casted into the wells of a paper substrate flushed with nitrogen and allowed to dry in the vacuum oven for 15 min. The Raman spectra of the analyte samples were collected across the entire well area. The experiments were repeated three times with different batches.

The SERS mapping technique was exploited to reveal the occurrence of molecular binding events between the SERS-sensitive area of the substrate and R6G. SERS signals were collected over a detection area of 30 × 30 μm with a laser spot size of 3 μm.

## Results and discussion

### Characterization of plasmonic nanohybridized paper substrate

The ability of block copolymers to form molecular assemblies in solution at the air-liquid interface is utilized to fabricate a thin organic film doped with silver nanoparticles (AgNPs) on the surface of the paper (Fig. 1a). Considering the porosity and surface roughness of the paper substrate, the morphology and distribution of the self-assembled AgNPs@film were studied by acquiring FE-SEM images (Fig. 1b-d). The FE-SEM micrographs revealed the formation of AgNPs with a size distribution of 47.5 nm and their close proximity to each other on the paper superficies.

**Fig. 2:**
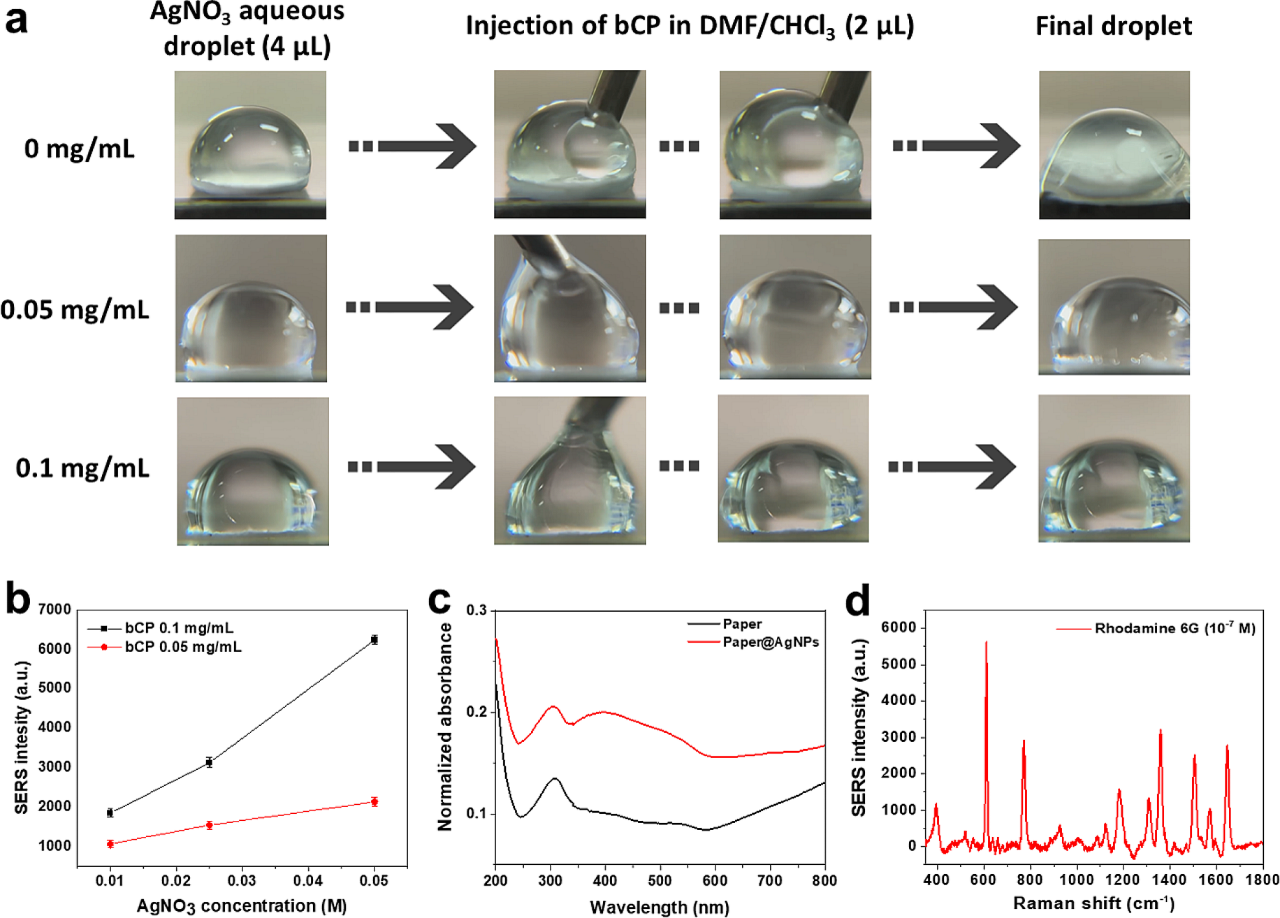
Effect of bCP concentration on microdroplet stability (**a**), components concentration-dependent variation in the SERS intensities (1 × 10^− 6^ M of R6G) (**b**), UV-Vis absorbance spectra of bare office paper and nanohybridized paper substrate (**c**), and SERS spectra of R6G on nanohybridized plasmonic paper substrate (**d**)

EDS mapping (Figure S1a and S1b) further confirmed the uniform distribution of the spherical AgNPs across the surface of the paper substrate in close proximity to each other. In addition, the more negligible porosity of the office paper (49%) resulted in the retention of particles close to the surface [38]. As anticipated, FE-SEM analysis of the nanohybridized plasmonic paper fabricated with lower concentrations of AgNO_3_ revealed lesser coverage of the paper surface with AgNPs, indicating that the concentration of precursors in a typical experiment is optimal for the pattern size (Figs. 1 and S1c-e). Interestingly, the lowest tested concentration of AgNO_3_ (0.01 M) resulted in the formation of non-uniformly aggregated nanoparticles on the surface of cellulose, whereas higher concentrations of AgNO_3_ resulted in a more even distribution and greater coverage of the surface of the cellulose fibers. Subsequently, we examined the effect of the bCP concentration on the formation of hybridized plasmonic paper, as shown in Fig. 2a and the supplementary video. Typically, the injection of DMF/CHCl_3_ containing bCP into the aqueous solution of AgNO_3_ resulted in mass transfer and self-assembly of the film with different structures imposed by the concentrations of the components. However, at the microdroplet scale, the variation of the bCP concentration directly influences the stability of the droplet. Specifically, the injection of a lower concentration of bCP decreased the stability of the droplets, whereas the injection of DMF/CHCl_3_ without bCP resulted in the immediate destruction of the droplet. FE-SEM images and droplet stability studies confirmed that the most suitable concentration of bCP and AgNO_3_ was 0.1 mg mL^− 1^ and 0.05 M, respectively. After treatment with NaBH_4_, the films were subjected to XRD analysis to ensure that the reduction of silver ions to AgNPs was successful, as shown in Figure S2. The cellulose lattice planes (110), (200), and (004) are responsible for the diffraction peaks at 2θ = 16.2°, 22.6°, and 35.9°, respectively. Weak diffraction peaks at 2θ = 29.7°, 36.1°, 39.6°, 43.3°, 47.8°, and 48.7° correspond to the (104), (110), (013), (020), (018), and (116) planes of CaCO_3_, which is found in standard office paper [25]. The peaks at 2θ = 44.4°, 64.5°, and 77.6° are ascribed to the (111), (200), (220), and (311) planes of the AgNPs, respectively.

To further investigate the most suitable ratio of components for the fabrication of nanohybridized plasmonic paper, we investigated the Raman enhancement for each substrate using R6G as the target analyte. As shown in Fig. 2b, the SERS intensity increases with increasing concentration of the precursors until it reaches a threshold beyond which the SERS signals disappear. For bCP, that limit was at the concentration of 0.1 mg mL^− 1^, at which the SERS intensity exceeded that of 0.05 mg mL^− 1^ more than threefold, whereas SERS signals were not obtained at 0.2 mg mL^− 1^. In this way, the optimal concentration of AgNO_3_, the precursor agent of the AgNPs, was determined to be 0.05 M. This can be attributed to the aggregation of AgNPs when a high concentration of AgNO_3_ is utilized, thus preventing the formation of hot spots. In contrast, the excess bCP might cover the surfaces of the AgNPs too thoroughly, thus restricting the attachment of R6G molecules to the AgNPs and preventing them from reaching the localized surface plasmon resonance areas. The particles appeared to be spherical and sporadically with small, truncated corners, which is likely owing to the involvement of bCP in the formation of the particles and the porous structure of the paper. This feature, in this regard, might be beneficial for SERS: the edged structure of metal nanoparticles leads to the more efficient formation of localized surface plasmons.

Classification of the developed substrate as reliable to enhance Raman signals requires the plasmon resonance spectrum to be separate from the charge transfer and absorption spectra. This is because plasmonic materials support electromagnetic SERS enhancement, whereas excitation via the chemical enhancement mechanism of charge transfer alone does not result in the conventional EF of SERS [28]. The absorption peak of the paper substrate on which the AgNPs is deposited is centered at about 400 nm, which is in accordance with data reported elsewhere for AgNPs (Fig. 2c). However, a broad absorption extending to higher wavelengths is contributed by the edged structure of the nanoparticles and is more closely associated with dipole plasmon resonance. Despite the possibility that the wavelength of the lasers used in the Raman experiments might cause fluorescence emission of the office paper samples, this fluorescence was quenched by the deposition of the AgNPs@bCP thin film. Thus far, this phenomenon has been explained in terms of Förster resonance energy transfer because of the presence of the deposited metal nanoparticles [38]. The deposited nanoparticles would also shield any fluorescence originating from the paper material.

The fact that electromagnetic enhancement plays a more significant role in SERS than chemical enhancement has been proven experimentally to date [28]. Successful SERS detection mainly depends on the interaction between the adsorbed molecules and plasmonic nanostructures. The presence of the pyridinic nitrogen atom in each bCP unit creates favorable conditions for the interaction of the substrate with analytes bearing any type of group capable of undergoing hydrogen bonding [39]. This feature was expected to contribute to achieving more prominent EFs inevitably.

### Stability and contact angle of microdroplet

**Fig. 3:**
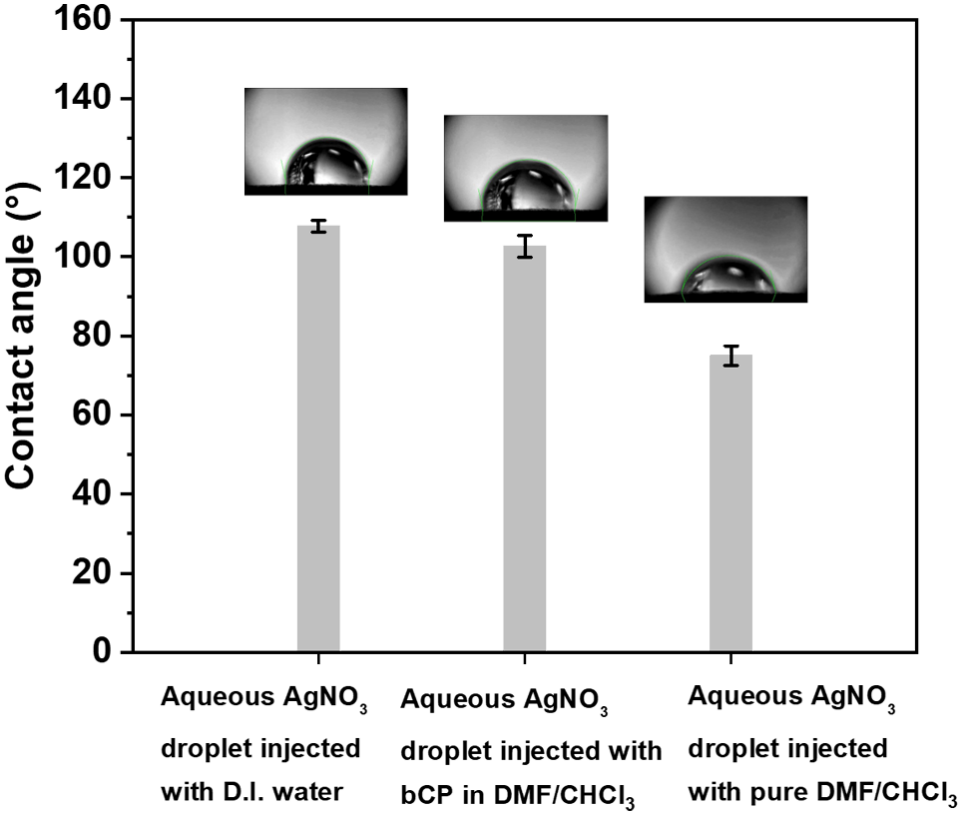
The contact angle of aqueous AgNO_3_ droplet (left) injected with DI water, (middle) injected with 0.1 mg mL^− 1^ bCP in DMF/CHCl_3_, and (right) injected with DMF/CHCl_3_. Inset: photographs of droplets corresponding to each case deposited on the wax-patterned paper

The stability of the microdroplet during the injection of bCP in DMF/CHCl_3_ was strongly dependent on the concentration of bCP. The effect of bCP on the stability of the microdroplet contact angle was further elucidated by comparing aqueous AgNO_3_ droplets injected with pure DMF/CHCl_3_ solution with those injected with bCP (Fig. 3). As a reference, an aqueous AgNO_3_ droplet injected with the same quantity of DI water was used. All three droplets were deposited on the wax-printed paper, which served as a hydrophobic surface. The reference droplets had an average contact angle of θ = 107.72°, whereas the droplet injected with DMF/CHCl_3_ had an average contact angle of θ = 74.97°. The observed decrease in the contact angle was induced by adding DMF/CHCl_3_ with a lower surface tension. The dissolution of bCP in DMF/CHCl_3_ increases the surface tension of the resulting blend, thus resulting in a droplet with a higher contact angle (Figs. 3 and S3). Moreover, the migration of bCP to the liquid/air interface allows the formation of a film that further stabilizes the microdroplet.

### SERS performances of the nanohybridized plasmonic paper substrate and spot-to-spot reproducibility

The morphological analysis confirmed that the significant Raman signal enhancement and good reproducibility are attributable to the favorable formation of the metal nanoparticles, which efficiently promote the generation of “hot spots” and propagation of surface plasmon resonances. As shown in the FE-SEM micrographs, along with the small interparticle distance, the presence of some irregular protrusions on the surfaces of the nanoparticles might contribute to the enhancement of the electromagnetic field. The SERS characteristics of the plasmonic paper substrate were investigated by using R6G (Fig. 2d). Overall, the characteristic Raman peak at 611 cm^− 1^ for R6G was selected to determine the EF, calculated to be 1.2 × 10^7^. However, to obtain a reliable sensor, the “hot spots” should be uniformly distributed to ensure that a stable Raman signal is detected from different locations.

**Fig. 4:**
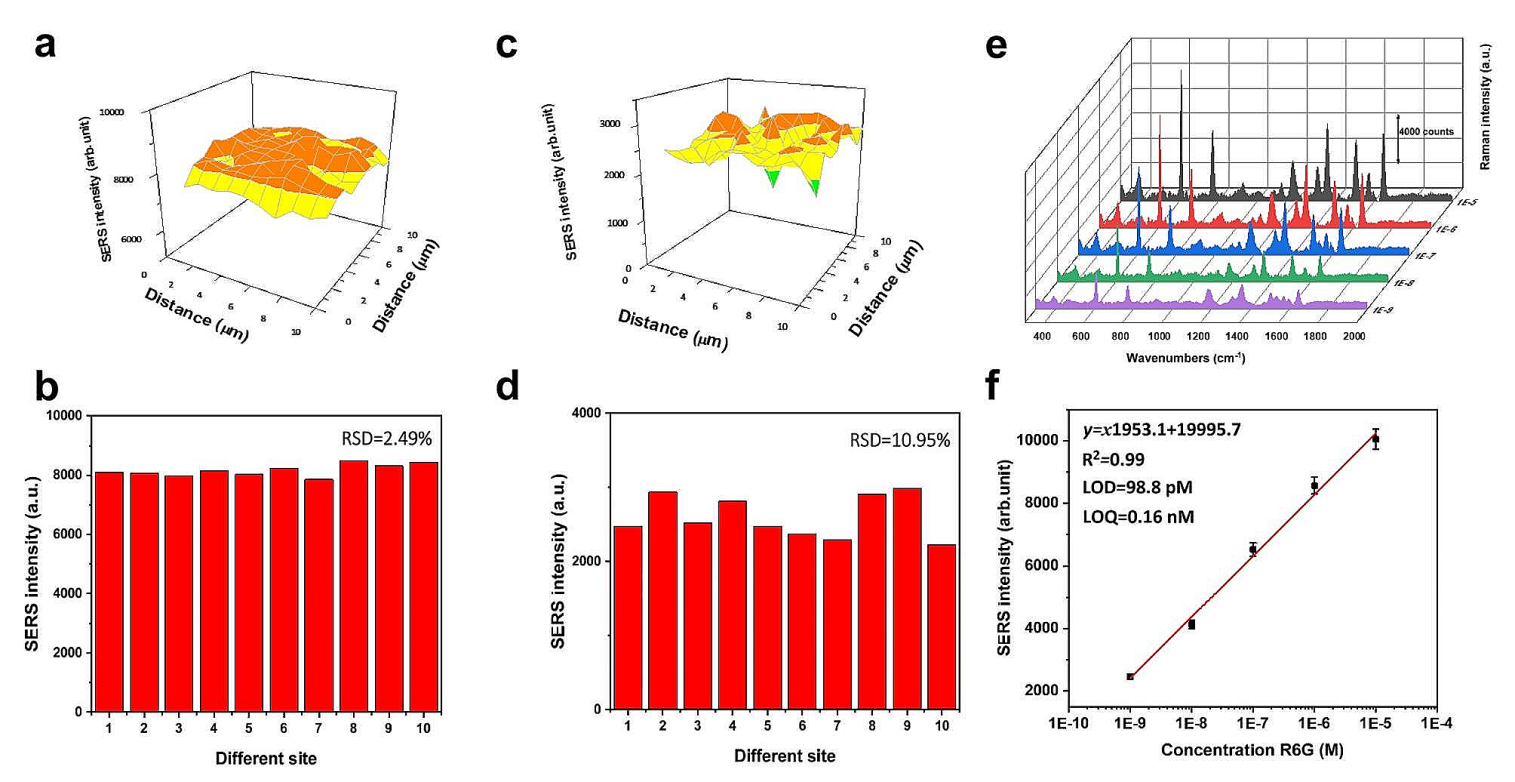
SERS mapping result of R6G at 611 cm^− 1^ extracted from 1 × 10^− 6^ M aqueous solutions (**a**,**b**) and from 1 × 10^− 9^ M aqueous solutions (**c**,**d**). RSD for aqueous solutions of rhodamine 6G (R6G; 1 × 10^− 6^ M and 1 × 10^− 9^ M) were 2.49% and 10.95%, respectively. SERS spectra of R6G obtained by varying the concentration of R6G (front to back: 10^− 9^, 10^− 8^, 10^− 7^, 10^− 6^, 10^− 5^ M) on the nanohybridized paper substrate (**e**). Relationship between the intensity of the band at 610 cm^− 1^ and the log concentration of R6G. Inset: linear equation, LOD, and LOQ (**f**)

Quantitative SERS detection can be achieved when the analyte concentration changes in a predictable manner [40]. This necessitated an examination of the distribution of the “hot spots” via SERS mapping with a laser spot size of ∼ 1 μm. This involved performing SERS measurements over a 10 × 10 μm area on the plasmonic nanohybridized paper substrate (Fig. 4a-d). The SERS mapping technique revealed notable signals at R6G concentrations of 1 × 10^− 6^ M and below. Furthermore, the probability of detecting noticeable SERS signals with 1 × 10^− 6^ M of R6G was determined to be equal across the substrate, indicating that the developed system is devoid of the notorious “coffee ring” effect associated with SERS substrates consisting of conventional paper (Fig. 4a). The random selection of ten spots to determine the uniformity of the SERS signal intensity led to the observation that the variance in data was not significant, with the RSD calculated to be 2.49% (Fig. 4b). As anticipated, the probability of observing stable SERS signals decreased when the concentration of R6G was lowered to 1 × 10^− 9^ M (Fig. 4c). At this concentration, much weaker SERS signals and significant fluctuation of the signal intensity were observed. As shown in Fig. 4d, the RSD of the SERS intensities of the 611 cm^− 1^ band of R6G (1 × 10^− 9^ M) among the ten random AgNP spots was 10.95%. To the best of our knowledge, the developed plasmonic paper has excellent spot-to-spot reproducibility for detecting R6G at a concentration of 1 × 10^− 6^ M.

### Sensitivity of nanohybridized plasmonic paper

To determine the sensitivity, SERS spectra of nanohybridized paper treated with 5 µL R6G droplets with concentrations ranging from 10^− 9^ to 10^− 6^ M were recorded on the portable Raman spectrometer, as shown in Fig. 4e. During the experiments, the major peaks were neither observed to shift nor did their Raman intensity change. The limit of detection (LOD) and limit of quantification (LOQ) of R6G using the nanohybridized paper sensor were estimated by conducting a linear fitting of the SERS intensities versus the log R6G concentration, as shown in Fig. 4f. The calibration curve has excellent linearity for the studied concentrations of R6G ranging from 10^− 9^ to 10^− 6^ M with a correlation coefficient of 0.99. The LOD and LOQ of R6G using the developed plasmonic paper sensor were estimated to be 48.9 pM and 0.16 nM, respectively.

The analytical performance of recently reported paper-based plasmonic substrates used for R6G detection was collected from the literature and compared to the results of the current study (Table 1). The sensitivity of our paper substrate is comparable to or exceeded that of previous research, but its spot-to-spot reproducibility is substantially superior.

**Table 1:** Comparison of SERS parameters with other paper-based plasmonic substrates

Substrates	Enhancement factor (EF)	LOD (M)	Spot-to-spot reproducibility (RSD)	Ref
AgNPs on A4 paper and AgNPs on Al foil	6.72 × 10^7^ and 1.03 × 10^8^		26.3% and 12.1%	[48]
Graphene oxide/ AgNPs on bacterial NFC nanopaper	-	0.13 × 10^− 9^	21%	[49]
AgNPs@filter paper	1.8 × 10^8^	1.2 × 10^− 9^	16.73%	[50]
Grape skin-AuNPs/GO based paper SERS	1.92 × 10^9^ for GE-AuNPs/GO and 5.8 × 10^4^ for GE-AuNPs	7.33 × 10^− 11^	9.6%	[51]
AgNPs inkjet printed on chitosan@paper	7.4 × 10^8^	1.07 × 10^− 11^	4.5%	[25]
Amphiphilic polymer self-assembled film containing AgNPs on office paper	1.2 × 10^7^	4.89 × 10^− 11^	2.49% for 1 × 10^− 6^ M R6G and 10.95% for 1 × 10^− 9^ M	This work

### Detection of SD and FLBN using nanohybridized plasmonic paper

**Fig. 5:**
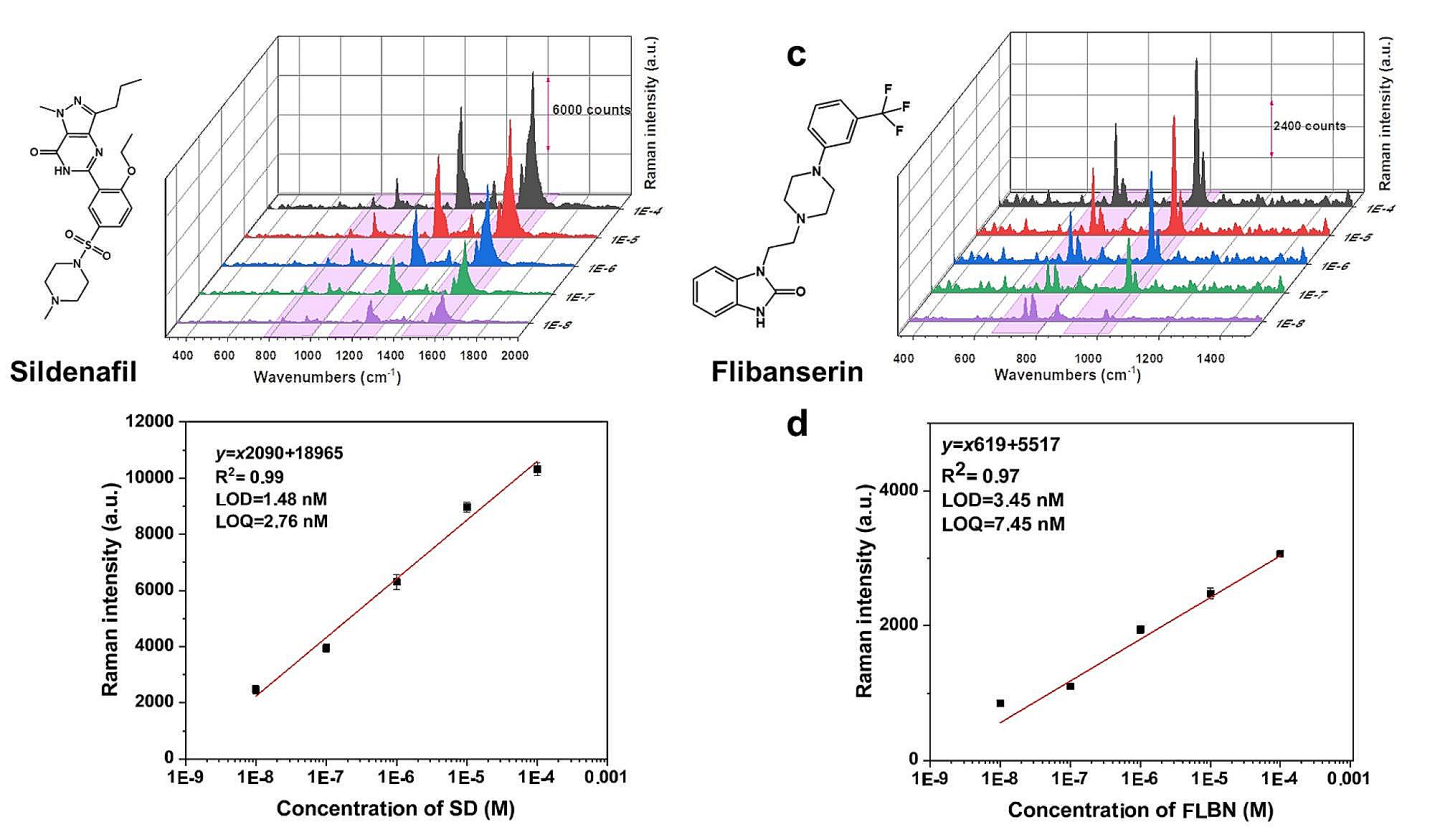
SERS spectra of SD **(a,b)** and FLBN **(c,d)** obtained by varying the concentration (front to back: 10^− 8^, 10^− 7^, 10^− 6^, 10^− 5^, 10^− 4^ M) on the nanohybridized paper substrate

As proof of concept, two emerging pollutants, SD and FLBN, were chosen as target analytes. SD is a phosphodiesterase-5 inhibitor (PDE-5i), which was originally developed to treat ischemic heart disease, pulmonary hypertension, and altitude sickness, but has since gained the reputation for treating erectile dysfunction (ED) [41, 42]. Illegal use as an ingredient [43] and the accessibility of generics [44], which are sold without a proper prescription, resulted in leakage into the water environment. Recently, Kim’s group reported that the concentrations of SD in local sewage treatment plants before and after purification were alarmingly high, especially on weekends [45]. Moreover, the results showed that the efficiency of biological nutrient removal (BNR) systems—the modified Ludzack-Ettinger (MLE; anoxic/oxic) and the A^2^/O (anaerobic/anoxic/oxic) processes—for the elimination of SD was alarmingly low. This reality prompted us to investigate the ability of the nanohybridized plasmonic paper we developed to quantitatively detect SD. We dropped 5 µL of solutions of SD with concentrations ranging from 10^− 4^ M to 10^− 8^ M in DI water onto the prepared substrate and recorded the Raman spectra, as shown in Fig. 5a. The characteristic Raman peaks of SD appeared with a slight shift in the SERS peaks compared to the Raman peaks of SD powder (Figure S7a and Table S1). As shown in Fig. 5b, the calibration curve exhibits excellent linearity for the tested SD concentrations ranging from 10^− 4^ to 10^− 8^ M, with a correlation value of 0.99. The estimated LOD and LOQ of SD utilizing the developed sensor were 1.48 nM and 2.76 nM, respectively.

On the other hand, FLBN is the only drug approved by the US Food and Drug Administration (USFDA) for treating hypoactive sexual desire (HASD), found in 7% of premenopausal women [46, 47]. The performance of the developed SERS substrate with respect to detecting FLBN was investigated by twice dropping and drying 5 µL of FLBN solutions with concentrations ranging from 10^− 4^ M to 10^− 8^ M, as shown in Fig. 5c. The characteristic Raman peaks of FLBN are detected with the SERS spectra slightly shifted compared to the Raman spectra of FLBN powder (Figure S7b and Table S2). The calibration curve of FLBN concentrations ranging from 10^− 4^ to 10^− 8^ M had good linearity with a correlation value of 0.97, as shown in Fig. 5d. The LOD and LOQ of FLBN using the nanohybridized plasmonic paper were estimated to be 3.45 nM and 7.45 nM, respectively.

In the evolving landscape of analytical techniques for pharmaceutical compounds, the developed sensing device based on SERS represents a significant advancement, particularly in the detection of FLBN and SD. The sensitivity of the developed SERS substrate demonstrated comparable, if not superior, performance relative to the established gold standard of chromatography-tandem mass spectrometry for analyzing FLBN, as shown in Table 2. Moreover, this method requires more complex setups and requires well-trained staff. On the other hand, other methods, such as spectrofluorimetry have also been used for sensing FLBN but showed a lower sensitivity, as shown in Table 2.

**Table 2:** Comparison of reported sensing methods for FLBN and SD

Method	Target	LOD (M)	Ref
HPLC-Mass spectrometry (MS)	FLBN	1.8 × 10^− 8^	[52]
HPLC- diode array detector (DAD)	FLBN	6.4 × 10^− 8^	
HPLC-Charger aerosol detector (CAD)	FLBN	1.3 × 10^− 6^	
Spectrofluorometric	FLBN	1.0 × 10^− 7^	[53]
Voltammetry	SD	5.5 × 10^− 8^	[54]

However, it’s important to acknowledge that our developed SERS substrate does not exhibit the same level of selectivity as some alternative SERS substrates modified for specific targets, notably those leveraging molecular imprinting or immunoassays. These techniques benefit from a high degree of specificity towards target molecules, a feature currently less pronounced in the developed SERS substrate. Despite this, the primary objective of this research was to pioneer novel, cost-effective, and simplified methodologies for SERS substrate fabrication based on the self-assembly of bCP in microdroplet.

## Conclusions

In summary, the self-assembly of block-copolymers (bCPs) at the air-liquid interface to form an organic film is an attractive feature. We took advantage thereof to develop an ultrasensitive detection method based on a SERS substrate in the form of a paper surface deposited with the film doped with silver nanospheres. An unprecedentedly low volume of components was utilized such that the entire fabrication process occurred on the scale of a single microdroplet stabilized by the bCP. FE-SEM micrographs revealed that, with the assistance of self-assembled copolymer film, AgNPs were formed with an average size of 47.5 nm and small, truncated corners. These morphological features of the AgNPs endowed the paper surface with plasmonic properties and leveraged the efficient generation of “hot spots.” The sensitivity of the designed nanohybridized plasmonic paper substrate was investigated by recording the SERS profiles of aqueous solutions of R6G. The generated electromagnetic field enhancement yielded an EF of 1.2 × 10^7^ and LOD of 48.9 pM for R6G. The analysis of two emerging pollutants, SD and FLBN, which was performed as proof of concept, showed that the nanohybridized plasmonic paper delivered excellent analytical performance. The LODs of SD and FLBN were estimated to be 1.48 nM and 3.45 nM, respectively. The novel approach has the advantage of allowing additional steps, such as synthesizing colloidal nanoparticles, to be bypassed during the preparation of the ultrasensitive and reproducible SERS substrate.

### Electronic supplementary material

Below is the link to the electronic supplementary material.

Supplementary Material 1

Supplementary Material 2

Supplementary Material 3

## Data Availability

The datasets used and/or analysed during the current study are available from the corresponding author on reasonable request.

## Abbreviations

AgNO_3_: silver nitrate
AgNP: silver nanoparticles
bCP: poly(styrene-b-2-vinyl pyridine) block copolymers
CHCl_3_: chloroform
DMF: dimethylformamide
ED: erectile dysfunction
EDS: Energy Dispersive Spectroscopy
EF: enhancement factor
FLBN: flibanserin
HASD: hypoactive sexual desire
LOD: limit of detection
LOQ: limit of quantification
NaBH_4_: sodium borohydride
PDE: 5i-phosphodiesterase-5 inhibitor
R6G: rhodamine 6G
SD: sildenafil
SERS: surface-enhanced Raman spectroscopy
USFDA: US Food and Drug Administration
XRD: X-ray diffraction
