# Supplementary material for: Highly sensitive plasmonic paper substrate fabricated via amphiphilic polymer self-assembly in microdroplet for detection of emerging pharmaceutical pollutants
Source: Nano Converg. 2024 Mar 29;11:13. doi: 10.1186/s40580-024-00420-x (PMC10980671; doi:10.1186/s40580-024-00420-x)
Supplement: Supplementary file 1 — Supplementary Material 1 [file 40580_2024_420_MOESM1_ESM.docx]

**SUPPORTING INFORMATION**

**Highly Sensitive Plasmonic Paper Substrate Fabricated via Amphiphilic Polymer Self-assembly in Microdroplet for Detection of Emerging Pharmaceutical Pollutants**

Mirkomil Sharipov^1,2,§^, Sarvar A. Kakhkhorov^1,§^, Salah M. Tawfik^3^, Shavkatjon Azizov^4^, Hong-Guo Liu^5^, Joong Ho Shin^6^, Yong-Ill Lee^1,4^*

^1^Anastro Laboratory, Institute of Basic Science, Changwon National University, Changwon 51140, Republic of Korea

^2^School of Mechanical Engineering, Yonsei University, Seoul 03722, Republic of Korea

^3^Department of Petrochemicals, Egyptian Petroleum Research Institute, Cairo 11727, Egypt

^4^Department of Pharmaceutical Sciences, Pharmaceutical Technical University, Tashkent 100084, Republic of Uzbekistan

^5^Key Laboratory for Colloid and Interface Chemistry of Education Ministry, Shandong University, Jinan 250100, P. R. China

^6^Division of Smart Healthcare, College of Information Technology and Convergence, Pukyong National University, Busan 48513, Republic of Korea

*______________________________________*

*Corresponding author: E-mail: yilee@changwon.ac.kr

^§^ These authors contributed equally to this work**.**

## ABSTRACT

We report an innovative and facile approach to fabricate an ultrasensitive plasmonic paper substrate for surface-enhanced Raman spectroscopy (SERS). The approach exploits the self-assembling capability of poly(styrene-b-2-vinyl pyridine) block copolymers to form a thin film at the air-liquid interface within the single droplet scale for the first time and the subsequent *in situ* growth of silver nanoparticles (AgNPs). The concentration of the block copolymer was found to play an essential role in stabilizing the droplets during the mass transfer phase and formation of silver nanoparticles, thus influencing the SERS signals. SEM analysis of the morphology of the plasmonic paper substrates revealed the formation of spherical AgNPs evenly distributed across the surface of the formed copolymer film with a size distribution of 47.5 nm. The resultant enhancement factor was calculated to be 1.2 × 10^7^, and the detection limit of rhodamine 6G was as low as 48.9 pM. The nanohybridized plasmonic paper was successfully applied to detect two emerging pollutants—sildenafil and flibanserin—with LODs as low as 1.48 nM and 3.45 nM, respectively. Thus, this study offers new prospects for designing an affordable and readily available, yet highly sensitive, paper-based SERS substrate with the potential for development as a lab-on-a-chip device.

**KEYWORDS:** surface-enhanced Raman scattering; air/liquid interface; block-copolymers; self-assembly; emerging pollutants

### 1. Enhancement factor

To calculate the enhancement factor (EF) of the developed plasmonic substrate, the Raman spectra of the 10-3 M aqueous solution of analytes were obtained. SERS signals were collected over a detection area of 30 × 30 µm with a laser spot size of 3 µm. Then, the SERS EFs were calculated using the following equation (1) and using the Raman intensity peak at 611 cm^-1^ for R6G:

|  | $EF=\frac{N_{reference} \times I_{plasmonic substrate}}{N_{plasmonic substrate} \times I_{reference}}$ | (1) |
| --- | --- | --- |

where I_reference_ and I_plasmonic substrate_ are the Raman intensities of 10^-3^ M of the analytes in the glass substrate and 1×10^-7^ M of the analytes on the plasmonic substrate, respectively. N_reference_ and N_plasmonic substrate_ are the total number of analytes molecules located in the laser spot area on the reference glass substrate and the plasmonic substrate, respectively. [1]

### 2. Limit of Detection and limit of quantification

The limit of detection (LOD) for R6G, sildenafil and flibanserin were calculated following the equation presented in previously reported works. [1,2] The SERS intensity versus the log concentration was linearly fitted, see equation (**1**).

Fitting equation:

$SERS intensity=a+b\times log(Concentration of analyte)$ (**1**)

*(a*: intercept; *b*: slope)

The criteria of LOD were used as 3σ + y0, and the SERS intensity of the blank sample was that of SERS sensor.

(σ: the standard deviation of SERS intensity of blank sample, y_0_: the SERS intensity of blank sample)

As a result, the LOD of R6G was calculated using equation (**2**).

$3\sigma+y0=a+b\times\log\left( LOD of analyte \right)$ (**2**)

$$LOD of analyte={10}^{[\frac{(3\sigma+y0)-a}{b}]}$$

### Figures


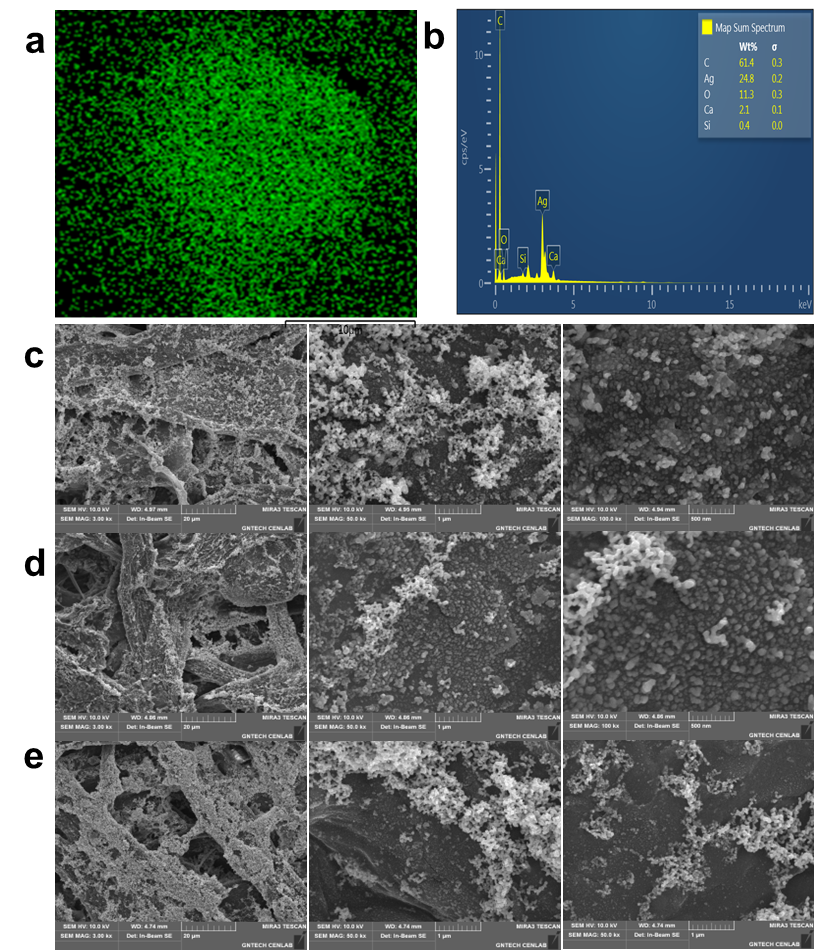


**Figure S1.** EDS mapping of nanohybridized plasmonic paper prepared with 0.05 M of AgNO_3_ (a-b). FE-SEM images of nanohybridized plasmonic paper fabricated with 0.05 M AgNO_3_ (c), 0.025 M AgNO_3_ (d), and 0.01 M AgNO_3_ (e).

**Figure S2**. X-ray diffraction pattern of nano-hybridized plasmonic paper.

**
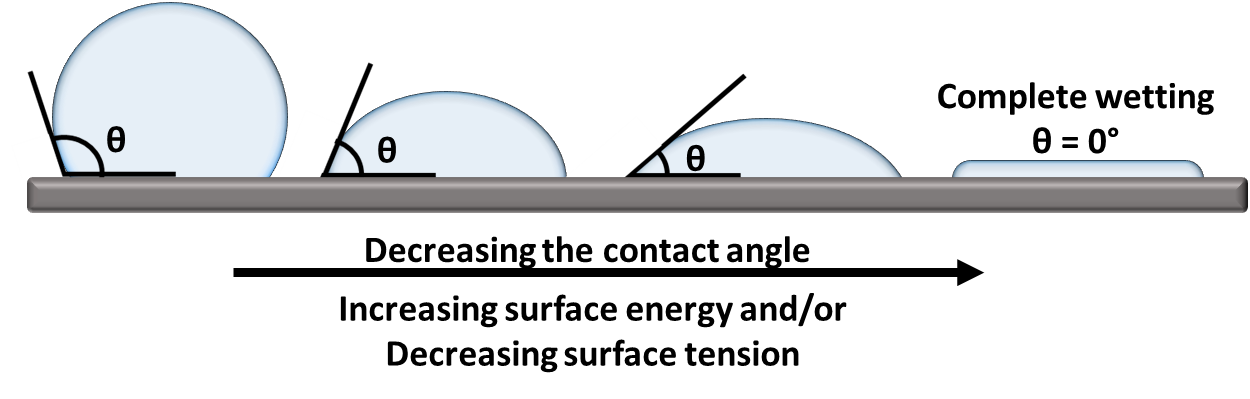
Figure S3.** Schematic illustration of the relation between contact angle and surface energy and/or surface tension.

**
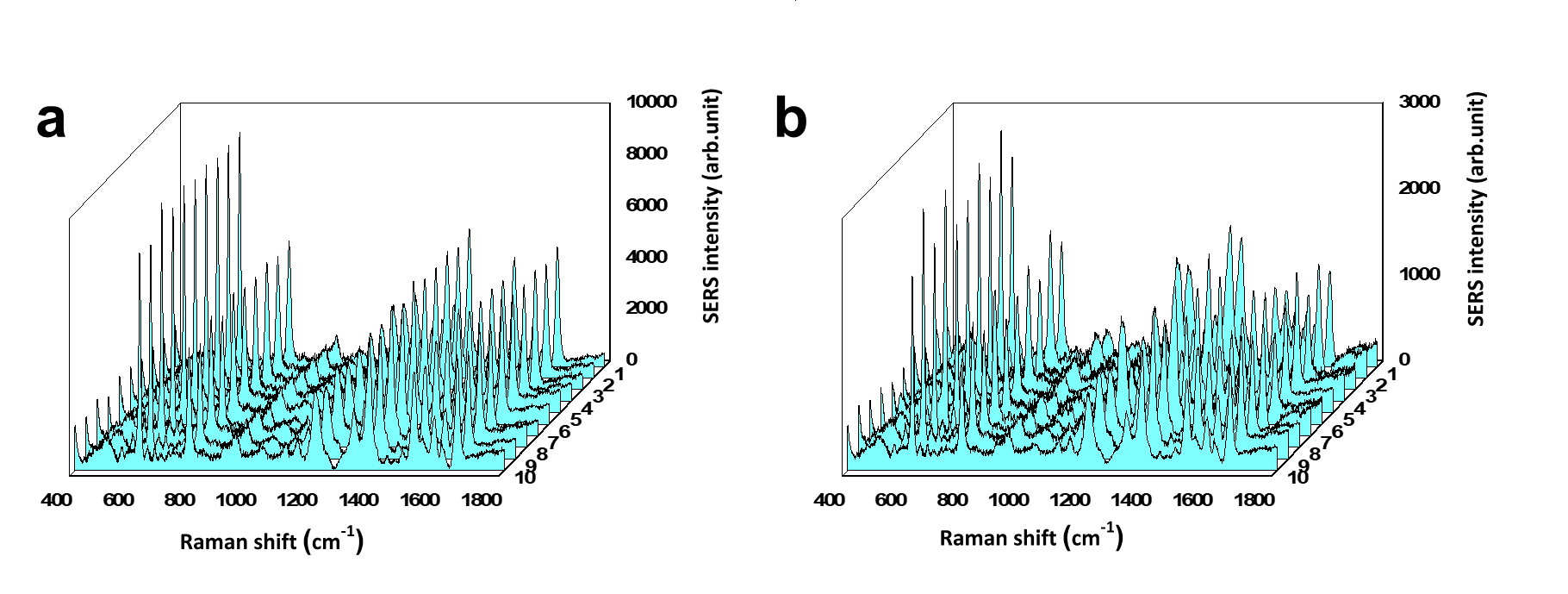
**

**Figure S4.** SERS mapping spectra of R6G at 611 cm^-1^ extracted from 1 × 10^-6^ M aqueous solutions (a) and from 1 × 10^-9^ M aqueous solutions (b).

**Figure S5.** SERS spectra of rhodamine 6G (R6G) obtained by varying the concentration of R6G ( from up to bottom: 10^-5^, 10^-6^, 10^-7^, 10^-8^, 10^-9^ M) on the nanohybridized paper substrate.

**Figure S6.** The stability of developed nanohybridized paper substrate over 20 days.

**
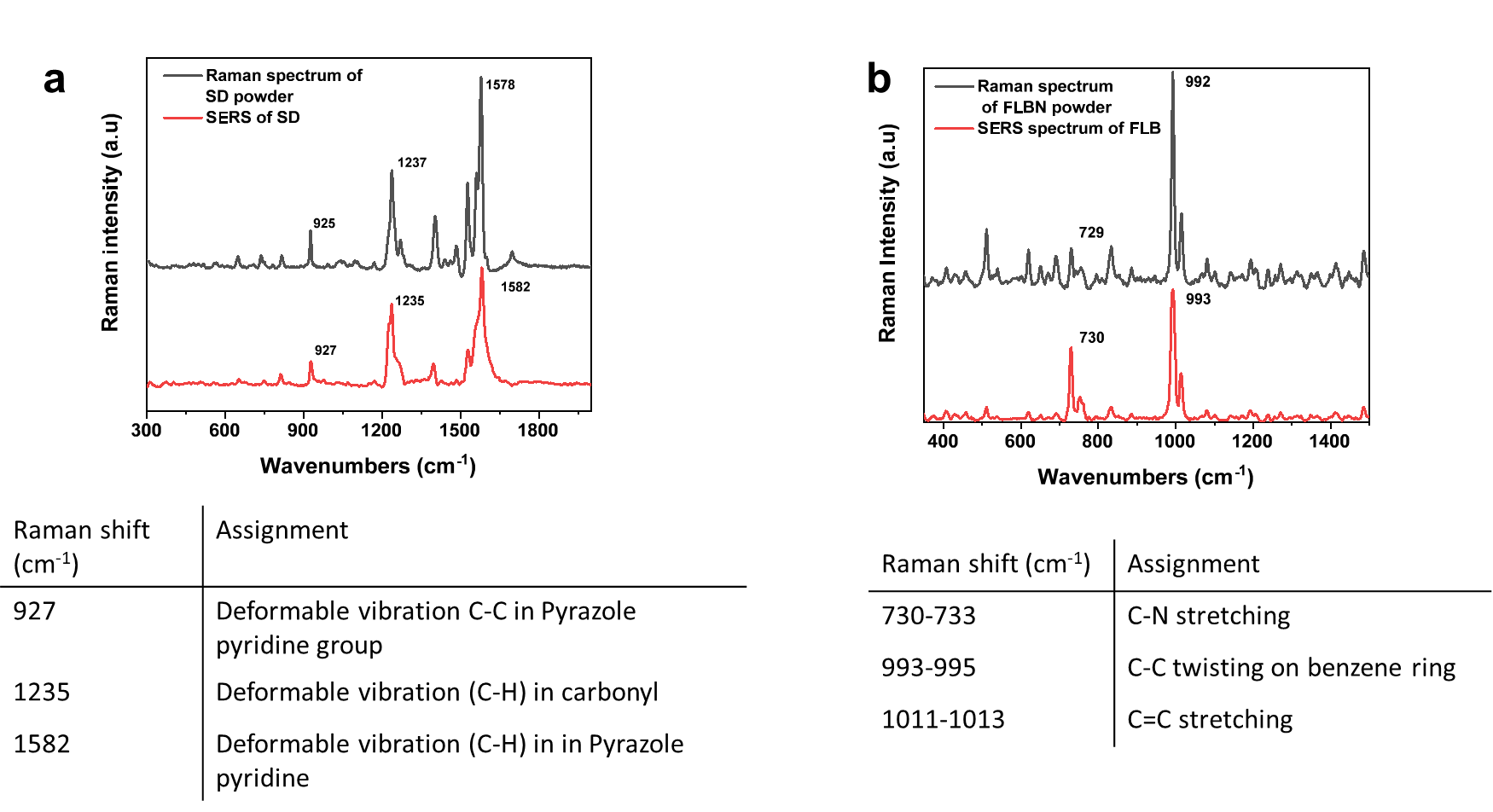
**

**Figure S7.** Comparison of SERS spectra to Raman spectra of SD and FLBN a and b, respectively. Tables show the characteristic Raman shift in SERS spectra of sildenafil [3-5] and flibanserin [6].

## REFERENCES

[1] T. T. Thuy, M. Sharipov, Y. Lee, B. T. Huy, Y.-I. Lee, Inkjet-based microreactor for the synthesis of silver nanoparticles on plasmonic paper decorated with chitosan nano-wrinkles for efficient on-site Surface-enhanced Raman Scattering (SERS), Nano Select 1 (5), (2020) p. 499‑509.DOI: [https://doi.org/10.1002/nano.202000081](about:blank)

[2] M. Lee, K. Oh, H.-K. Choi, S. G. Lee, H. J. Youn, H. L. Lee, D. H. Jeong, Subnanomolar Sensitivity of Filter Paper-Based SERS Sensor for Pesticide Detection by Hydrophobicity Change of Paper Surface, ACS Sens. 3 (1), (2018) p. 151‑159.DOI: [https://doi.org/10.1021/acssensors.7b00782](about:blank)

[3] H. Zhao, W. Hasi, L. Bao, S. Han, X. Sha, J. Sun, X. Lou, D. Lin, Z. Lv, Rapid Detection of Sildenafil Drugs in Liquid Nutraceuticals Based on Surface-Enhanced Raman Spectroscopy Technology, Chin. J. Chem . 35 (10), (2017) p. 1522‑1528.DOI: [https://doi.org/10.1002/cjoc.201700168](about:blank)

[4] D. T. C. Minh, L. A. Thi, N. T. T. Huyen, L. Van Vu, N. T. K. Anh, P. T. T. Ha, Detection of sildenafil adulterated in herbal products using thin layer chromatography combined with surface enhanced Raman spectroscopy: “Double coffee-ring effect” based enhancement, J. Pharm. Biomed. Anal. 174, (2019) p. 340‑347.DOI: [https://doi.org/10.1016/j.jpba.2019.05.043](about:blank)

[5] L. Lin, F. Qu, P. Nie, H. Zhang, B. Chu, Y. He, Rapid and Quantitative Determination of Sildenafil in Cocktail Based on Surface Enhanced Raman Spectroscopy, Molecules (Basel, Switzerland) 24 (9), (2019) p. 1790.DOI: [https://doi.org/10.3390/molecules24091790](about:blank)

[6] Q. Bao, H. Zhao, S. Han, C. Zhang, W. Hasi, Surface-enhanced Raman spectroscopy for rapid identification and quantification of Flibanserin in different kinds of wine, Anal. Methods 12 (23), (2020) p. 3025‑3031.DOI: [https://doi.org/10.1039/D0AY00741B](about:blank)
