# Supplementary figures and images for: Highly sensitive plasmonic paper substrate fabricated via amphiphilic polymer self-assembly in microdroplet for detection of emerging pharmaceutical pollutants
Source: Nano Converg. 2024 Mar 29;11:13. doi: 10.1186/s40580-024-00420-x (PMC10980671; doi:10.1186/s40580-024-00420-x)

## Slide 1
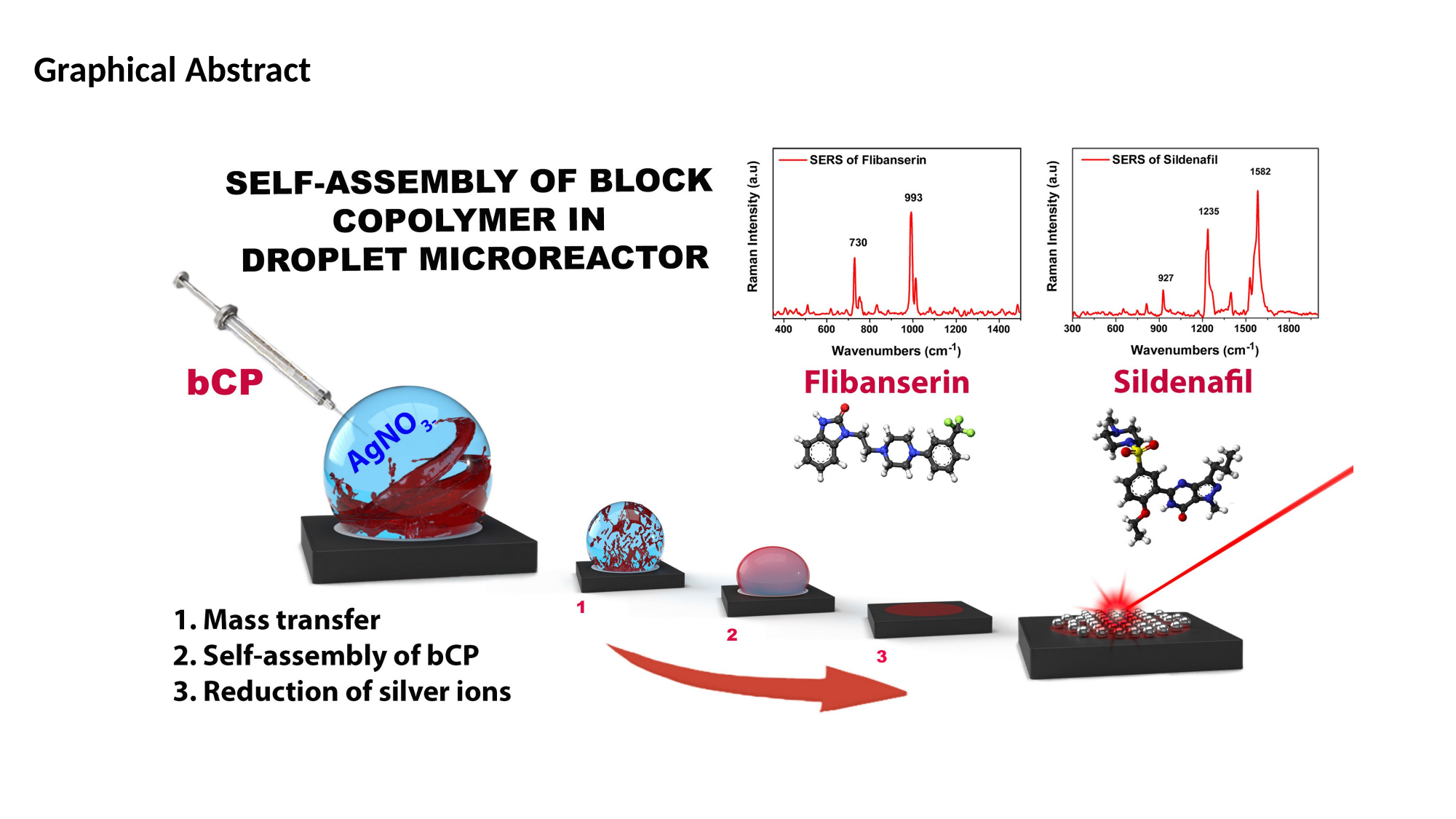

Graphical Abstract

Supplement: Supplementary file 3 — Supplementary Material 3 [file 40580_2024_420_MOESM3_ESM.pptx]
